# Supplementary material for: Molecular Genetic Diversity and Combining Ability for Some Physiological and Agronomic Traits in Rice under Well-Watered and Water-Deficit Conditions
Source: Plants (Basel). 2022 Mar 5;11(5):702. doi: 10.3390/plants11050702 (PMC8912379; doi:10.3390/plants11050702)
Supplement: Supplementary file 1 [file plants-11-00702-s001.zip › plants-1594147-supplementary.pdf]

Table S1. Physical and chemical soil characteristics of the experimental sites during 2020 and 2021 growing seasons.

**Characteristics**	2020	2021
Soil particles distribution		
Sand (%)	15.33	14.80
Silt (%)	33.20	32.50
Clay (%)	51.47	52.70
Soil texture	Clay	Clay
pH (1: 2.5 water suspension)	8.03	8.17
EC (dS m^{-1})	2.30	2.56
Organic matter	1.63	1.56
Soluble cations and anions (mmolc L^{-1})		
Ca^{2+}	10.70	10.0
Mg^{2+}	4.20	3.96
K^{+}	1.92	1.78
Na^{+}	13.9	13.04
HCO_3^{-}	13.50	15.60
Cl^{-}	8.60	7.30
SO_4^{2-}	8.70	7.50
CO_3^{2-}	0.0	0.0

Table S2. List of SSR primers and their sequences used in this study.

No.	Marker	Forward primer	Reverse primer
1	RM315	GAGGTACTTCCTCCGTTTCAC	AGTCAGCTCACTGTGCAGTG
2	RM543	CTGCTGCAGACTCTACTGCG	AAATATTACCCATCCCCCCC
3	RM263	CCCAGGCTAGCTCATGAACC	GCTACGTTTGAGCTACCACG
4	RM279	GCGGGAGAGGGATCTCCT	GGCTAGGAGTTAACCTCGCG
5	RM55	CCGTCGCCGTAGTAGAGAAG	TCCCGGTTATTTTAAGGCG
6	RM518	CTCTTCACTCACTCACCATGG	ATCCATCTGGAGCAAGCAAC
7	RM159	GGGGCACTGGCAAGGGTGAAGG	GCTTGTGCTTCTCTCTCTCTCTCTCTCTC
8	RM3805	AGAGGAAGAAGCCAAGGAGG	CATCAACGTACCAACCATGG
9	RM70	GTGGACTTCATTTCAACTCG	GATGTATAAGATAGTCCC
10	RM234	ACAGTATCCAAGGCCCTGG	CACGTGAGACAAAGACGGAG
11	RM72	CCGGCGATAAAACAATGAG	GCATCGGTCCTAACTAAGGG
12	RM223	GAGTGAGCTTGGGCTGAAAC	GAAGGCAAGTCTTGGCACTG
13	RM160	CGTCGGATGATGTAAAGCCT	CATATCGGCATTCGCCTG
14	RM222	CTTAAATGGGCCACATGCG	CAAAGCTTCCGGCCAAAAG
15	RM332	GCGAAGGCGAAGGTGAAG	CATGAGTGATCTCACTCACCC
16	RM20A	ATCTTGTCCCTGCAGGTCAT	GAAACAGAGGCACATTTCATTG

Table S3. Separate analysis of variance of all the studied traits under each environment.

Source of Variance		Days to Heading				Plant Height			
		First Season		Second Season		First Season		Second Season	
	DF	Normal	Stress	Normal	Stress	Normal	Stress	Normal	Stress
Genotypes	20	128.28**	123.40**	138.97**	152.96**	442.89**	468.29**	449.91**	440.94**
GCA	5	216.12**	180.88**	232.91**	277.66**	988.82**	1193.55**	1079.75**	1103.62**
SCA	15	99.00**	104.24**	107.66**	111.39**	260.91**	226.54**	239.96**	220.05**
Error	40	0.95	0.9	1.25	0.92	4.58	5.04	5.53	4.62

Source of Variance		Leaf Rolling				Relative Water Content			
		First Season		Second Season		First Season		Second Season	
	DF	Normal	Stress	Normal	Stress	Normal	Stress	Normal	Stress
Genotypes	20	0.60**	4.89**	0.65**	4.58**	21.91**	117.35**	43.55**	132.19**
GCA	5	0.58*	5.94**	0.77**	9.77**	25.63**	186.50**	44.84**	179.70**
SCA	15	0.63**	4.54**	0.61**	2.86**	20.67**	94.30**	43.12**	116.36**
Error	40	0.22	0.27	0.11	0.13	1.39	1.2	1.05	1.54

Source of Variance		Chlorophyll Content				Number of Panicles/Plant			
		First Season		Second Season		First Season		Second Season	
	DF	Normal	Stress	Normal	Stress	Normal	Stress	Normal	Stress
Genotypes	20	18.30**	28.39**	20.25**	25.28**	47.80**	17.60**	33.71**	14.37**
GCA	5	14.30**	18.99**	12.31**	23.55**	60.85**	11.47**	33.89**	8.25**
SCA	15	19.64**	31.53**	22.90**	25.86**	43.45**	19.64**	33.65**	16.41**
Error	40	0.93	1.01	1.28	0.98	1.12	0.83	0.86	0.68

Source of Variance		Number of Filled Grains/Panicle				Sterility Percentage			
		First Season		Second Season		First Season		Second Season	
	DF	Normal	Stress	Normal	Stress	Normal	Stress	Normal	Stress
Genotypes	20	383.96**	411.72**	647.15**	717.54**	19.59**	43.50**	40.97**	35.68**
GCA	5	305.76**	447.79**	553.12**	669.30**	13.70**	49.69**	41.74**	36.93**
SCA	15	410.03**	399.69**	678.50**	733.62**	21.55**	41.44**	40.72**	35.27**
Error	40	30.56	25.13	29.21	33.05	0.33	0.8	0.47	0.87

Source of Variance		1000-Grain Weight				Grain Yield/Plant			
		First Season		Second Season		First Season		Second Season	
	DF	Normal	Stress	Normal	Stress	Normal	Stress	Normal	Stress
Genotypes	20	7.72**	4.29**	8.06**	3.96**	114.62**	63.66**	66.18**	73.71**
GCA	5	3.99**	1.50*	5.48**	1.99*	152.51**	112.24**	95.83**	122.08**
SCA	15	8.96**	5.31**	8.93**	4.62**	101.99**	47.47**	56.29**	57.59**
Error	40	0.55	0.59	0.62	0.77	2.39	1.82	2.29	2.23

Table S4. General combining ability effects (GCA) of the evaluated parents for all studied traits under each environment.

Trait	Env.	Parents						LSD (gi) 0.05	LSD (gi) 0.01
		P_1	P_2	P_3	P_4	P_5	P_6		
DTH	E1	-3.87**	-2.25**	-1.59**	2.21**	1.62**	3.87**	0.37	0.49
	E2	-3.78**	-1.14**	-1.71**	1.57**	1.12**	3.94**	0.35	0.47
	E3	-3.93**	-2.49**	-1.58**	2.48**	1.55**	3.98**	0.42	0.57
	E4	-4.94**	-2.33**	-1.13**	2.12**	2.03**	4.25**	0.36	0.48
	Comb.	-4.13**	-2.05**	-1.50**	2.09**	1.58**	4.01**	0.18	0.24
PH	E1	-4.27**	-5.70**	-5.77**	7.42**	0.14	8.18**	0.81	1.08
	E2	-5.68**	-6.20**	-4.67**	9.51**	-1.01*	8.05**	0.85	1.13
	E3	-5.20**	-6.11**	-4.88**	10.00**	0.12	6.07**	0.89	1.19
	E4	-5.64**	-6.37**	-4.00**	8.41**	-0.78	8.38**	0.81	1.08
	Comb.	-5.20**	-6.09**	-4.83**	8.83**	-0.38	7.67**	0.41	0.54
LR	E1	0.06	-0.11	-0.07	0.20*	0.11	-0.18*	0.18	0.24
	E2	0.09	0.81**	-0.18	-0.42**	0.25*	-0.54**	0.21	0.28
	E3	0.16*	-0.17**	-0.17**	0.12	0.21**	-0.14*	0.12	0.16
	E4	1.07**	0.30**	-0.71**	-0.48**	0.05	-0.23**	0.11	0.15
	Comb.	0.34**	0.21**	-0.28**	-0.15**	0.15**	-0.27**	0.08	0.10
RWC	E1	-0.59*	0.41	0.18	1.68**	-1.36**	-0.32	0.44	0.59
	E2	-2.00**	-3.05**	3.16**	3.28**	-2.08**	0.68**	0.41	0.55
	E3	-0.29	-0.22	-0.73**	0.93**	-1.82**	2.12**	0.39	0.52
	E4	-2.08**	-3.40**	2.41**	3.06**	-1.78**	1.79**	0.47	0.62
	Comb.	-1.24**	-1.57**	1.25**	2.24**	-1.76**	1.07**	0.21	0.28
CHLC	E1	0.53**	0.92**	-0.81**	0.18	-1.06**	0.23	0.36	0.49
	E2	0.52**	0.19	0.45*	-0.18	-1.70**	0.71**	0.38	0.51
	E3	0.62**	0.52*	-0.86**	0.04	-0.90**	0.59**	0.43	0.57
	E4	0.86**	0.43*	-0.99**	0.41*	-1.49**	0.78**	0.37	0.50
	Comb.	0.64**	0.51**	-0.55**	0.11	-1.29**	0.58**	0.19	0.25
NP	E1	-0.18	-0.11	1.39**	1.87**	-2.67**	-0.30	0.40	0.53
	E2	0.16	-0.44*	0.40*	0.62**	-1.19**	0.44*	0.34	0.46
	E3	-0.56**	-0.22	1.66**	1.19**	-1.45**	-0.63**	0.35	0.47
	E4	-0.41*	-0.09	1.02**	0.24	-0.65**	-0.11	0.31	0.41
	Comb.	-0.25**	-0.21*	1.12**	0.98**	-1.49**	-0.15	0.17	0.23
NFG	E1	-0.10	-1.02	6.16**	1.56	-3.86**	-2.73*	2.08	2.79
	E2	-2.68**	-3.01**	6.40**	4.12**	-4.34**	-0.48	1.89	2.53
	E3	-2.80**	0.41	7.64**	2.63*	-1.61	-6.28**	2.04	2.72
	E4	-5.13**	-3.48**	8.48**	4.03**	-0.32	-3.57**	2.16	2.90
	Comb.	-2.68**	-1.78**	7.17**	3.08**	-2.53**	-3.27**	1.00	1.32
SP	E1	-0.34**	0.02	-1.18**	0.10	1.12**	0.28*	0.22	0.29
	E2	1.04**	0.79**	-1.09**	-1.65**	1.93**	-1.02**	0.34	0.45
	E3	-1.30**	-0.89**	-1.10**	0.18	1.28**	1.83**	0.26	0.35
	E4	0.87**	0.62**	-1.18**	-1.29**	1.72**	-0.74**	0.35	0.47
	Comb.	0.07	0.13	-1.14**	-0.66**	1.51**	0.09	0.14	0.19
	E1	0.59**	0.40**	-0.06	-0.43**	-0.32*	-0.18	0.28	0.37
	E2	0.13	-0.04	-0.04	-0.18	-0.24	0.37*	0.29	0.39
TGW	E3	0.80**	-0.42**	0.16	-0.37*	-0.36*	0.20	0.30	0.40
	E4	0.05	-0.49**	0.01	0.30	-0.12	0.25	0.33	0.44
	Comb.	0.39**	-0.14	0.02	-0.17*	-0.26**	0.16*	0.15	0.19
GYPP	E1	2.05**	0.52	2.21**	1.30**	-4.13**	-1.94**	0.58	0.78
	E2	-1.55**	-3.12**	1.75**	2.78**	-0.41	0.54*	0.51	0.68
	E3	0.84**	1.73**	2.24**	-0.24	-2.87**	-1.71**	0.57	0.76
	E4	-1.54**	-2.41**	3.01**	2.19**	-1.76**	0.51	0.56	0.75
	Comb.	-0.05	-0.82**	2.30**	1.51**	-2.29**	-0.65**	0.27	0.36

*and ** indicate p-value < 0.05 and 0.01, in the same order

DTH is days to heading, PH is plant height, CHLC is chlorophyll content (SPAD reading), LR is leaf rolling, RWC is relative water content, NP is number of panicles per plant, SP is sterility percentage, TGW is 1000-grain weight (g) and GYPP is grain yield per plant (g).

Table S5. Specific combining ability effects (SCA) for the 15 F_1 hybrids for studied traits under each environment.

Genotype	DTH					PH					LR					RWC				
	E1	E2	E3	E4	Comb.	E1	E2	E3	E4	Comb.	E1	E2	E3	E4	Comb.	E1	E2	E3	E4	Comb.
P1×P2	-1.65**	-0.57	-0.01	-2.01**	-1.18**	7.08**	12.96**	8.32**	13.17**	10.38**	-0.17	-0.40	-0.13	-0.03	-0.18	3.75**	2.72**	4.14**	2.52**	3.28**
P1×P3	-0.31	1.45**	-0.20	-8.18**	-3.39**	1.35	2.22	3.70**	0.91	2.05**	-0.27	-0.24	-0.20	-0.85**	-0.39**	3.17**	2.09**	3.63**	1.49*	2.60**
P1×P4	5.89**	1.74**	5.75**	2.70**	0.21	1.16	-0.58	-0.93	-3.28**	-0.91	-0.01	-0.16	0.04	-1.30**	-0.36**	-0.58	4.58**	-1.07*	3.65**	1.64**
P1×P5	-9.19**	-10.67**	-10.36**	-0.64	0.07	-15.83**	-14.72**	-10.79**	-14.08**	-13.85**	1.11**	1.50**	1.31**	0.56**	1.12**	-3.40**	0.67	-4.59**	5.42**	-0.48
P1×P6	6.90**	7.83**	6.18**	-0.35	0.92**	-1.40	-0.44	0.79	1.08	0.01	-0.11	0.26	-0.19	-1.19**	-0.31**	-1.76**	-5.09**	-5.25**	-5.95**	-4.51**
P2×P3	5.41**	4.81**	2.33**	2.24**	3.57**	0.01	-1.88	-4.72**	0.20	-1.60**	0.94**	3.20**	-0.12	0.09	1.03**	-4.15**	-4.02**	-4.16**	-3.75**	-4.02**
P2×P4	5.28**	6.04**	6.58**	-4.55**	-2.00**	-0.44	-5.39**	2.29	-1.88	-1.36*	0.27	-0.39	0.49**	0.02	0.10	-2.51**	-0.94	-1.47**	-1.59*	-1.63**
P2×P5	6.53**	7.32**	7.54**	0.78	2.94**	16.20**	-0.39	13.52**	-1.75	6.90**	-0.17	-0.61*	-0.14	-0.05	-0.24*	1.35*	7.59**	2.49**	7.42**	4.71**
P2×P6	-6.38**	-7.83**	-6.92**	0.07	0.63*	-9.40**	-4.60**	-2.87*	-2.85*	-4.93**	-0.03	-0.92**	0.05	-0.87**	-0.44**	-0.25	6.63**	-2.21**	6.05**	2.56**
P3×P4	3.95**	3.10**	4.36**	0.95	0.88**	10.63**	14.22**	17.08**	13.89**	13.95**	-0.21	-1.19**	0.04	0.04	-0.33**	2.77**	2.90**	3.08**	2.07**	2.70**
P3×P5	5.87**	3.86**	5.29**	0.28	-2.93**	11.09**	14.26**	10.31**	13.61**	12.32**	-0.12	-1.07**	-0.05	-0.48**	-0.43**	1.50*	3.58**	2.11**	2.80**	2.50**
P3×P6	0.62	0.56	3.19**	-2.76**	-1.33**	-6.74**	-6.02**	-4.33**	-11.27**	-7.09**	0.42	0.23	0.54**	0.29	0.37**	-3.42**	-3.85**	-6.51**	-4.84**	-4.65**
P4×P5	-3.92**	-3.22**	-4.80**	-4.85**	-2.08**	-13.55**	-7.25**	-9.95**	-7.80**	-9.64**	0.01	1.00**	0.06	1.44**	0.63**	-0.90	-9.81**	-0.04	-11.32**	-5.52**
P4×P6	-4.85**	-6.06**	-5.89**	-2.55**	-2.14**	2.80*	1.20	0.10	3.04**	1.78**	0.00	0.07	0.10	-0.77**	-0.15	2.51**	1.09	0.88	6.96**	2.86**
P5×P6	1.40**	4.58**	1.43*	-3.22**	-2.04**	4.57**	-1.78	-4.90**	3.23**	0.28	-0.01	1.55**	-0.09	1.29**	0.68**	2.82**	-8.91**	-0.12	-10.35**	-4.14**
LSD Sij 0.05	1.01	0.97	1.17	1.09	0.6	2.21	2.32	2.43	2.22	1.12	0.49	0.57	0.33	0.31	0.21	1.22	1.13	1.06	1.28	0.57
LSD Sij 0.01	1.35	1.30	1.56	1.45	0.8	2.96	3.11	3.25	2.97	1.48	0.65	0.76	0.44	0.41	0.28	1.63	1.51	1.42	1.71	0.76

Genotype	CHLC					NP					NFG					SP				
	E1	E2	E3	E4	Comb.	E1	E2	E3	E4	Comb.	E1	E2	E3	E4	Comb.	E1	E2	E3	E4	Comb.
P1×P2	1.10*	0.60	1.38*	0.84	0.98**	4.81**	1.64**	2.84**	1.35**	2.66**	7.54*	3.59	0.40	4.36	3.97**	-2.42**	-3.63**	-0.94*	-3.22**	-2.55**
P1×P3	0.75	0.52	0.56	-0.42	0.35	5.21**	1.93**	4.39**	1.43**	3.24**	7.16*	7.60**	10.05**	6.76*	7.89**	-0.02	2.93**	0.68	2.96**	1.64**
P1×P4	1.35**	3.38**	1.11	0.60	1.61**	-0.96	0.21	-0.27	3.43**	0.60*	-8.39**	8.98**	3.38	-2.58	0.35	-0.52	0.54	0.32	0.11	0.11
P1×P5	-3.42**	-0.53	-3.43**	-2.41**	-2.45**	-0.42	3.17**	3.22**	2.39**	2.09**	-7.14*	-	-	-	-14.00**	3.85**	2.23**	5.06**	2.37**	3.38**
P1×P6	2.26**	-0.26	1.11	0.87	1.00**	-4.26**	-3.04**	-4.67**	-2.98**	-3.74**	-5.56	-6.44*	6.16*	0.06	-1.45	4.03**	3.35**	1.18**	3.00**	2.89**
P2×P3	-2.23**	-5.53**	-3.18**	-4.30**	-3.81**	-6.25**	-4.18**	-2.54**	1.42**	-2.89**	-26.06**	-	-	-	-21.75**	5.14**	2.50**	7.08**	2.53**	4.31**
P2×P4	-2.76**	-4.67**	-4.67**	-2.93**	-3.76**	-3.85**	-1.24*	-3.69**	-2.36**	-2.78**	-10.57**	-	-	-	-19.58**	2.31**	6.22**	5.35**	5.80**	4.92**
P2×P5	1.05*	3.53**	1.83**	1.96**	2.09**	2.15**	3.34**	0.03	2.28**	1.95**	18.26**	20.27**	17.91**	19.14**	18.89**	-0.62*	0.29	-0.95**	0.43	-0.21
P2×P6	1.89**	1.87**	2.16**	-0.46	1.37**	5.37**	-0.32	4.61**	-1.47**	2.05**	5.90*	6.26*	20.81**	18.27**	12.81**	0.81**	-2.57**	0.14	-2.92**	-1.13**
P3×P4	4.64**	3.62**	4.81**	5.95**	4.76**	3.01**	2.87**	1.85**	1.04*	2.19**	8.49**	5.13	14.13**	7.91*	8.92**	0.21	-1.27**	-2.09**	-0.88	-1.00**
P3×P5	1.47**	-0.06	1.39*	-0.94	0.46	-4.07**	-3.88**	-6.19**	-1.43**	-3.89**	4.81	-	10.95**	4.42	2.15	-1.26**	-1.29**	-1.20**	-1.22*	-1.24**
P3×P6	-0.09	2.42**	-0.02	-0.12	0.55*	-1.36*	2.05**	0.97*	1.12*	0.69**	-3.12	-5.83*	-9.58**	-	-7.86**	0.05	0.47	3.28**	0.05	0.96**
P4×P5	1.99**	-0.66	2.09**	-3.11**	0.08	3.97**	-2.67**	2.83**	-3.12**	0.25	9.30**	7.66**	8.07**	14.92**	9.99**	-1.85**	1.94**	1.55**	1.55**	0.80**
P4×P6	-0.96	3.71**	0.60	0.49	0.96**	1.55**	1.75**	2.05**	2.30**	1.91**	11.97**	11.67**	5.05	13.36**	10.51**	-1.44**	-5.00**	0.21	-1.96**	-2.05**
P5×P6	-4.26**	-4.97**	-4.25**	-4.08**	-4.39**	0.50	-0.11	-0.33	2.03**	0.52*	-13.94**	-	-5.84*	-	-14.84**	2.09**	6.70**	0.86*	6.38**	4.01**
LSD Sij 0.05	1.00	1.04	1.17	1.02	0.52	1.10	0.94	0.96	0.85	0.47	5.72	5.18	5.59	5.95	2.74	0.59	0.92	0.71	0.96	0.40
LSD Sij 0.01	1.33	1.39	1.56	1.37	0.68	1.47	1.26	1.28	1.14	0.62	7.65	6.94	7.48	7.95	3.63	0.79	1.24	0.95	1.29	0.52

Table S5 (Cont). Specific combining ability effects (SCA) for the 15 F_1 hybrids for all 1000-grain weight and grain yield per plant under each environment.

Genotype	TGW					GYPP				
	E1	E2	E3	E4	Comb.	E1	E2	E3	E4	Comb.
P1×P2	1.29**	-0.30	1.75**	1.04*	0.94**	4.33**	0.55	1.94*	2.64**	2.36**
P1×P3	1.10**	1.61**	0.51	0.48	0.93**	-2.87**	-0.99	2.45**	-2.12**	-0.88*
P1×P4	-2.14**	-2.34**	-0.90*	-0.34	-1.43**	2.37**	3.74**	3.81**	4.55**	3.62**
P1×P5	-3.11**	-0.74	-2.52**	1.03*	-1.34**	-4.54**	2.80**	-6.19**	4.99**	-0.73
P1×P6	1.91**	0.54	1.98**	-0.24	1.05**	-0.53	-2.77**	1.12	-1.61*	-0.95*
P2×P3	-1.50**	-1.64**	-2.31**	-0.16	-1.40**	-16.34**	-8.76**	-4.70**	-4.92**	-8.68**
P2×P4	-0.13	-0.42	0.71	0.07	0.06	-3.56**	-3.45**	-1.39	-3.43**	-2.96**
P2×P5	0.50	1.00*	1.33**	0.84	0.92**	7.66**	5.74**	7.91**	6.19**	6.88**
P2×P6	0.02	0.87*	-0.70	1.38**	0.39	-0.42	4.35**	-1.33	4.84**	1.86**
P3×P4	2.65**	1.33**	2.31**	1.05*	1.83**	6.99**	2.58**	4.43**	4.01**	4.50**
P3×P5	2.17**	0.19	2.52**	-1.55**	0.83**	3.50**	-1.13	2.30**	-0.07	1.15**
P3×P6	-2.05**	0.60	0.86*	1.07*	0.12	3.11**	2.72**	-7.76**	1.73*	-0.05
P4×P5	0.96*	0.73	0.62	0.66	0.74**	-0.91	1.34	-0.74	-5.08**	-1.35**
P4×P6	0.64	1.02*	-0.21	0.29	0.43*	0.22	1.90**	0.35	2.82**	1.32**
P5×P6	-1.16**	-2.66**	-1.90**	-2.74**	-2.11**	-3.56**	-4.41**	3.31**	-7.06**	-2.93**
LSD Sij 0.05	0.77	0.80	0.81	0.90	0.40	1.60	1.40	1.57	1.54	0.74
LSD Sij 0.01	1.03	1.07	1.09	1.21	0.53	2.14	1.87	2.10	2.07	0.99

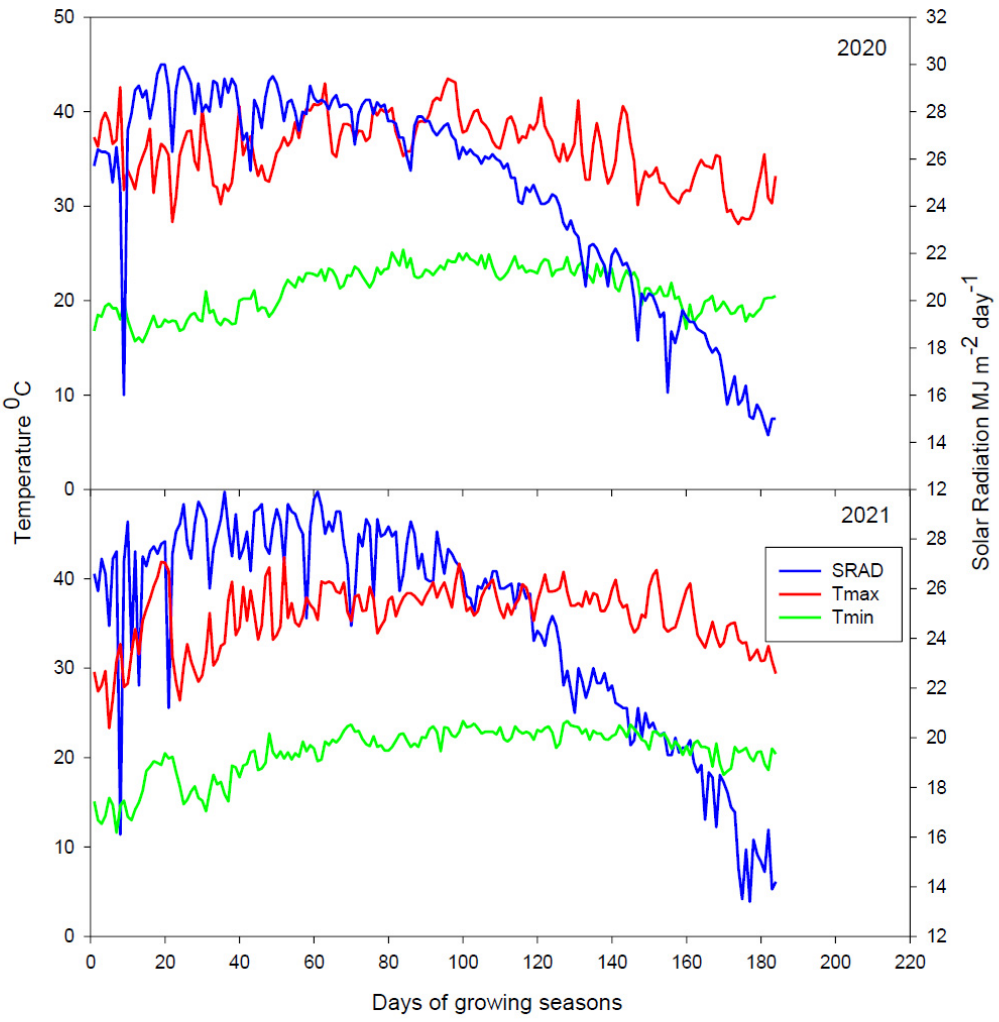

Figure S1. Certain meteorological data at the experimental site in the two summer seasons of 2020 and 2021.

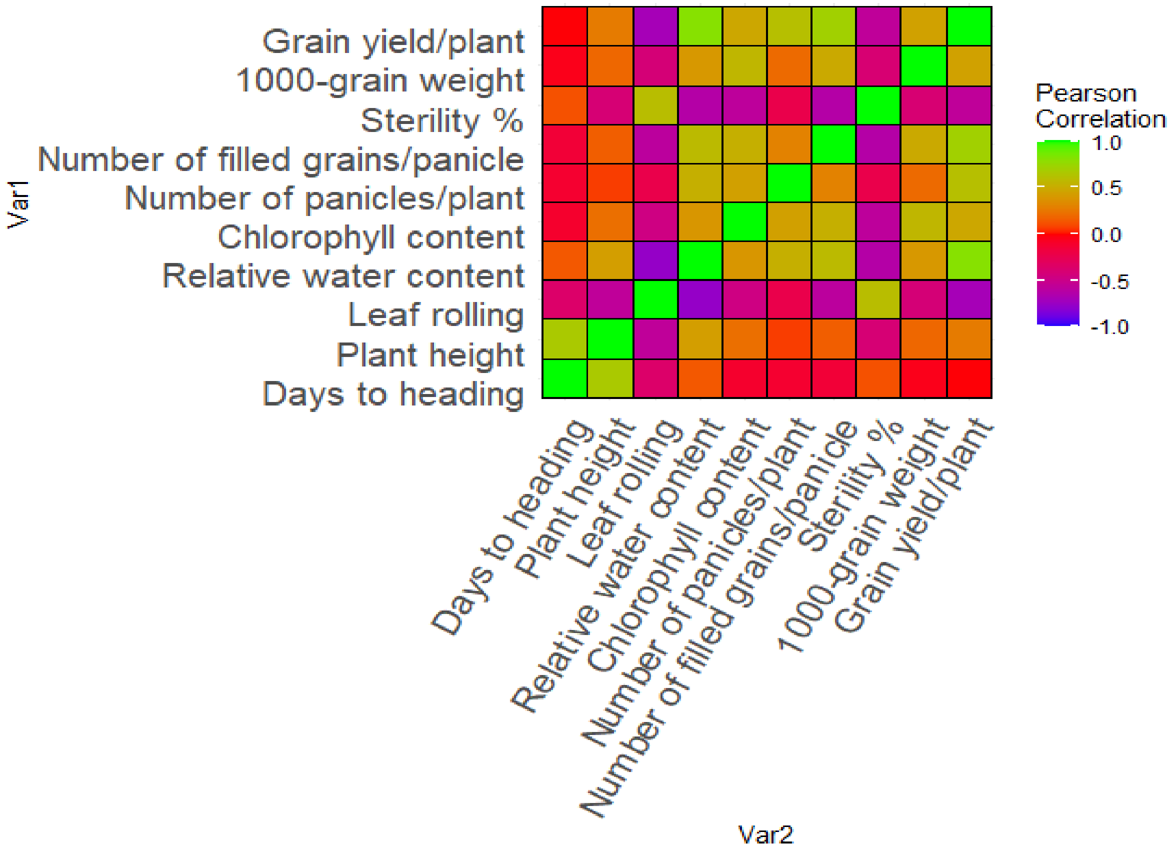

Figure S2. Correlation heatmap of the evaluated traits under water deficit conditions.
